# Potential Ecological Risk and Characterization of Floating Microplastics in the Surface Water of a Highly Urbanized Large River in Southeast Asia

**DOI:** 10.1155/sci5/3043345

**Published:** 2025-10-08

**Authors:** Mohammad Abdul Momin Siddique, Samsun Naher, Nazrul Islam, Md. Yeamin Hossen, Sayma Tabassum, Azad Uddin, Koushik Das, M. Safiur Rahman

**Affiliations:** ^1^Department of Oceanography, Noakhali Science and Technology University, Noakhali 3814, Bangladesh; ^2^Faculty of Fisheries and Protection of Waters, South Bohemian Research Center of Aquaculture and Biodiversity of Hydrocenoses, Research Institute of Fish Culture and Hydrobiology, University of South Bohemia in Ceske Budejovice, Zatisi 728/II, Vodnany 38925, Czech Republic; ^3^Water Quality Research Laboratory, Chemistry Division, Atomic Energy Center, Bangladesh Atomic Energy Commission, 4-Kazi Nazrul Islam Avenue, Dhaka 1000, Bangladesh

**Keywords:** Bangladesh, ecological risk, freshwater, Jamuna River, microplastics, plastic pollution, polymers

## Abstract

Microplastics (MPs) are widespread in aquatic environments and pose significant threats to ecosystems, wildlife, and human health. While MP pollution in water has received considerable attention, there is still limited understanding of its regional distribution, shapes, and associated risks. The Jamuna River, one of the largest freshwater ecosystems in Bangladesh, is increasingly contaminated by hazardous MPs, likely originating from industrial, domestic, hospital waste, and municipal sewage. This study aimed to investigate the abundance, distribution, characteristics, and ecological risks of MPs in the surface water of a highly urbanized large river in Southeast Asia. Water samples were collected from 30 sites along a 44 km stretch of the river during two periods: March to April (premonsoon) and July to August (monsoon). A total of 386 MPs were identified in the collected samples. The mean abundance of MPs in the surface water of the Jamuna River ranged from 0.01 to 0.15 MPs/L during the premonsoon season and from 0.01 to 0.13 MPs/L during the monsoon season. Approximately 79.78%–87.98% of the particles fell within the 100–1500 μm size range. The presence of polyethylene (31.60%–37.24%), polystyrene (PS) (6.71%–10.81%), and polyvinyl chloride (PVC) (5.64%–7.28%) contributed significantly to MP contamination, posing a high health hazard. Several risk assessment models were employed to evaluate the associated risks. The pollution load index (PLI) classified the contamination under risk Category I (minor pollution). Meanwhile, the polymer hazard index (PHI) values were 305.65 for the premonsoon season and 208.0 for the monsoon season, both indicating hazard Category IV (“danger” level). The potential ecological risk index (PERI) placed the Jamuna River surface water in the “extreme danger” risk category, with values of 2454.43 during the premonsoon and 1911.29 during the monsoon. The prevalence of high-hazard polymers such as PVC and PS suggests a potential risk of toxicological effects for aquatic organisms and humans through food web transfer. This baseline study provides valuable insights into the MP pollution of one of the country's major rivers. It may help improve our understanding of land-based MP inputs from inland water bodies severely impacted by anthropogenic activities.

## 1. Introduction

The pervasiveness and detrimental properties of microplastics (MPs) have made them a global issue [1]. The increasing production and disposal of synthetic plastic products in freshwater and marine environments have raised serious concerns worldwide. Because of growing populations and rates of consumption, plastic production rose from 1.7 million tons in 1950 to 400.3 million tons in 2022 [2]. Plastics are widely used in many different industries because of their affordable, lightweight, and chemically stable characteristics [1]. Plastics persist in aquatic environments due to limited ultraviolet (UV) penetration and stable conditions, which slow down their degradation. MPs typically reach the marine ecosystem from terrestrial settings by direct dumping, drainage, wind movement, and inappropriate disposal. One of the many sources of MPs is the breakdown and fragmentation of plastic bottles, bags, rope, pipes, and other waste plastics through surface water runoff [3, 4]. MPs can accumulate over long periods in lakes, rivers, and the marine environment due to their high persistence and buoyancy. Since aquatic waterbodies are frequently found in valleys and low-lying areas, freshwater ecosystems are the paramount destination for many pollutants entering the watershed. Most of the efforts in MP research focused on marine environments, while less than 4% of studies were associated with freshwaters [5]. Recently, different studies have been performed to evaluate the occurrence, abundance, and impacts of MPs in the marine environment [6–9] and the freshwater environment [10, 11]. However, studies on the occurrence, abundance, and characterization of MPs in the surface water of rivers are limited in the literature.

Rivers are the primary source of land-based MPs, representing approximately 80% of total plastic debris input into the world's oceans [12]. According to numerous studies, being close to densely inhabited areas, industry, wastewater treatment facilities, tributaries, and similar factors may be linked to higher MP abundance in water and river sediments [13]. Through the food web, various species in aquatic environments, including plankton and higher vertebrates, consume MPs [14]. When MPs are consumed, aquatic organisms' digestive systems may become physically blocked, causing starvation, stunted growth, and reproductive issues [15]. Furthermore, MPs act as vectors for pollutants; they can absorb harmful chemicals like persistent organic pollutants (POPs) and heavy metals from the surrounding water, which are then introduced into the food chain when ingested by aquatic life [16]. This bioaccumulation and biomagnification of toxins can have cascading effects, ultimately impacting higher trophic levels, including humans [15]. The long-term ecological consequences of MP pollution are profound and far-reaching, disrupting ecosystems, threatening biodiversity, and posing a significant challenge to environmental conservation efforts.

Bangladesh has approximately 700 rivers and tributaries and is a highly populated country. Land-based plastic debris is the primary source of MP pollution in the river systems. Every day, between 4000 and 4500 tons of solid garbage are produced, with over half of that amount being dumped into freshwater or low-lying land [17]. One of the primary sources of pollution in Bangladesh is poorly managed plastics. Recently, the country was placed 10th out of the 20 countries that produce the most mismanaged plastic garbage globally [16]. The freshwater ecosystems in Bangladesh are vulnerable to environmental problems due to MP contamination [18]. The issue has been worsened by Bangladesh's quick industrialization and urbanization, which have increased the amount of plastic debris that gets into the waterways [19]. MPs have been found in major rivers in Bangladesh, including the Meghna River [20], Karnafully River [21], Turag River [14], and Buriganga River [18, 22]. These rivers are vital to the livelihood and health of millions of people [4]. Although MP contamination has been reported in several Bangladeshi rivers (e.g., Meghna, Karnafully, Turag, Buriganga), no baseline data exist for the Jamuna River, one of the largest freshwater systems in South Asia. Unlike previously studied rivers, the Jamuna's hydrology, discharge, and role as a primary conduit to the Bay of Bengal present unique ecological implications for regional and marine MP transport. Furthermore, by applying risk assessment indices (polymer hazard index [PHI], pollution load index [PLI], and potential ecological risk index [PERI]) in a high-flow tropical river system, this study provides new insights into how polymer composition and hydrological seasonality influence ecological risk levels.

The Jamuna River is one of the largest rivers in the country. It flows through many cities, which provides benefits to local populations, and irrigation, energy generation, navigation, recreation, fisheries, and numerous other industrial and domestic uses heavily rely on river water. Every year, large amounts of fish are caught from the Jamuna River, accounting for 6445 metric tons [23]. Despite the importance of this river, these aquatic systems are facing a variety of threats. They are heavily polluted, as several industrial zones have been established near the bank of the Jamuna River, and the toxic effluents, including trace metals, pesticides, and organic and inorganic pollutants from these industries, are directly entering the river. Besides, domestic and agricultural effluents and airborne deposition can be possible sources of MP pollution in the surface water of the Jamuna River. However, no studies have been conducted to investigate the MPs contamination level in the surface water of the Jamuna River. Therefore, the present study aimed to investigate the occurrence, abundance, and characteristics of MP in the surface water, their spatial and seasonal variation, and their ecological risk assessment in the Jamuna River. The study is anticipated to offer significant baseline data on the quantity and characteristics of MPs, which will be helpful for upcoming professionals involved in biodiversity protection and ecological health monitoring.

## 2. Materials and Methods

### 2.1. Sampling Area and Sample Collection

**Figure 1:**
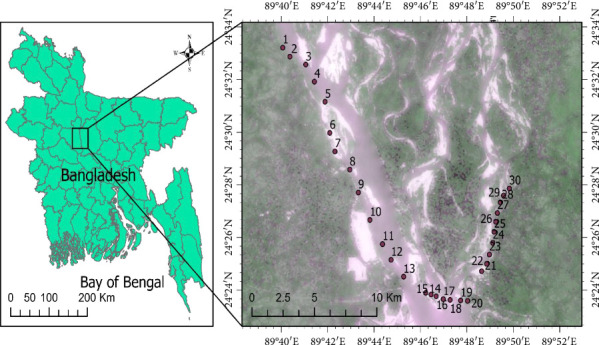
Location map of the study area of the Jamuna River, Bangladesh.

A total of 30 sampling stations were selected on the left and right banks of the Jamuna River during the premonsoon (March–April) and monsoon season (July–August). Sampling locations were selected based on site characteristics and pollution load, primary land use, and the possibility of sampling. For collecting water samples, 1000 mL of surface water (from 0 to 12-cm depth) was gently sieved and filtered through a 63 μm mesh-sized stainless-steel sieve, and the residue on the sieve was stored in a 500 mL glass bottle, which was appropriately washed with distilled water before sampling. Two replicates were maintained for each sampling site. Although replication was limited to two per station due to logistical constraints, duplicate sampling ensured cross-validation, and strict QA/QC measures minimized variability. Then, the water samples were put in an icebox and returned to the Department of Oceanography, Noakhali Science and Technology University laboratory for further analysis. The geographic locations of the sampling locations in the Jamuna River are shown in Figure 1, and the sampling information on the geographical position system (GPS) for each sampling point of the Jamuna River systems can be found in the supporting section (Table S1).

### 2.2. Sample Preparation and Extraction of MPs

A 500 mL water sample was digested in the laboratory with 25 mL H_2_O_2_ for biological materials. To enhance the digestion, the beakers were covered with aluminum foil and placed in an incubator (Velp Inc., Italy) at 60°C for an hour. Following incubation, MPs were separated from the undesirable substances in the water by floatation using a sodium iodide (NaI) solution (4.4 M, 1.5 g/mL) (Merck, Germany) [24]. The supernatant was gently collected in a collection beaker and filtered with a 0.45 μm cellulose nitrate filter (Sartorius GmbH, Germany) using a vacuum pump (V300 Wiggens, Germany). It should be noted that using a 63 μm sieve may underestimate the abundance of smaller MPs (< 100 μm), which were beyond the detection capacity of this study. All filters were kept on glass Petri dishes after being dried at ambient temperature (32°C). The filter samples were sent to the Water Quality Research Laboratory of the Atomic Energy Centre, Dhaka (AECD), to arrange polymer analysis.

### 2.3. Identification and Quantification of MPs

A Zeiss stereomicroscope Stemi 508 magnification was used to acquire pictures of plastic particles, and a light binocular microscope (Labomed, model: CXL-110446002, 9135002) was used to examine the specimens at × 4 and × 10 magnification visually. The maximum length, size, color, and shape of the samples were examined and documented [25]. Based on their shape and availability, the particles were divided into the following categories: fragment, foam, fiber, and pellet. Additionally, they were divided into seven different color groups: red, transparent, green, blue, black, and grey. The size of MPs was also divided into three groups: Class 1: 100–1500 μm, Class 2: 1500–3000 μm, and Class 3: 3000–5000 μm. Every sample's MP count, color, and size were meticulously recorded.

### 2.4. Polymer Identification by FTIR

Fourier transform infrared FTIR spectroscopy (Thermo Fisher Scientific Nicolet iN10, USA) in transmittance mode was used to identify the polymer type in plastic-like particles. Due to resource constraints, FTIR analysis was conducted on a representative subset (10–12 particles per filter). While this is consistent with many MP studies, it may introduce underrepresentation of less abundant polymer types. FTIR, a technique that generates spectra with distinct bands, is ideal for small samples and provides molecular-level plastic characterization [26]. Thermo Fisher's OMNIC Picta software was utilized for size measurement, processing, and IR spectrum analysis. The detector offered a resolution of 4 cm^−1^, 32 co-scans, and a spectral range of 4000–400 cm^−1^. Particles were classified as MPs if the quality index was 75% or higher, based on comparison with the reference OMNIC spectra library [27]. Particles with a matching degree and quality index between 65% and 75% were further analyzed and evaluated by comparing their absorption frequencies to the known polymer spectral database.

### 2.5. Quality Assurance and Quality Control

In this experiment, glassware, stainless steel tools, and containers were used. All lab equipment was rinsed with ethanol and Milli-Q water, then dried on a clean bench. Mixing, digestion, and filtering were done in a fume hood to prevent airborne contamination [25]. Cotton lab coats and polymer-free gloves were worn at all times during the study. If samples were not immediately analyzed, they were covered by aluminum foil to avoid contamination. Every step, from collection to final analysis, was conducted with care to prevent cross-contamination, including sample transportation, storage, digestion, filtration, and microscopic and FTIR analysis. Two samples (*n* = 2) were examined from each location for reliable results. A separate reagent was used for blank/control samples to avoid contamination. Blank controls were analyzed alongside each block of 10 water samples. No MPs were detected in blanks; therefore, reported abundances were not adjusted. Pure cotton lab attire and nitrile–butadiene gloves were worn throughout the experiment to minimize background contamination. For each block of water samples (10 sets), one blank sample was analyzed to check for contamination during sample preparation and filtering.

### 2.6. MP Risk Assessment Approach

The polymer hazard scores and polymer types were used as indices to assess the risks of MPs [28], as follows:

(1) $$\begin{array}{c} \text{PHI}=\sum P_{n}\times S_{n}, \end{array}$$

where *P*_*n*_ = % of each MP polymer type at each sampling location, and *S*_*n*_ = score for the polymers comprising the MPs. The values of polypropylene (PP), polyethylene terephthalate (PET), polystyrene (PS), polyethylene (PE), and polyvinyl chloride (PVC) were 1, 4, 30, 11, and 10001, respectively [28].

The PLI is classified into four hazard categories and is calculated using the following formula [29]:

(2) $$\begin{array}{c} \text{PLI}=\sqrt{\left(\frac{C_{i}}{C_{0}}\right)}, \end{array}$$

where *C*_*i*_ = abundance of each MP in the surface water samples. *C*_0_ = baseline abundance of MPs, the lowest mean value obtained from the sampling sites (equal to 0.01 MPs/L).

To evaluate the ecological risks, several preliminary evaluation techniques were used. The following is the calculation of a modified assessment model for possible ecological risk:

(3) $$\begin{array}{c} \text{Potential ecological risk factors}\left(E_{i\text{⁣}}\right)=T_{i}\times \left(\frac{C_{i}}{C_{0}}\right), \end{array}$$

(4) $$\begin{array}{c} P\text{ERI}=\overset{n}{\underset{i=1}{\sum }}E_{i}, \end{array}$$

where *T*_*i*_ is the chemical toxicity coefficient for the constituent polymer [28] and *C*_*i*_/*C*_0_ is a quotient for the observed MP concentration versus the background level. Due to a lack of available background data, the lowest MP concentration in the surface water measured in this study (0.01 MPs/L) was adopted as the background value. The criteria for the risk levels were based on the PHI, PLI, and PERI, shown in Supporting Table S2.

It should be noted that the hazard scores used in PHI and PERI calculations were derived from Lithner et al. [28]. While this framework remains widely adopted, more recent ecotoxicological assessments (e.g., [30, 31]) may provide updated hazard characterizations. Future studies should integrate these approaches for a more nuanced evaluation.

### 2.7. Data Analysis

MP abundance data were presented as mean ± standard deviation. The distribution pattern of the MP data, which suggests a normal distribution, was examined using the Shapiro–Wilk normality test. In order to comprehend the seasonal change in the MP abundance of several stations, a Student's *t*-test was conducted. For all analyses, the alpha threshold for significant differences was set at 0.05. A paired group (UPGMA-unweighted) cluster analysis was performed to group similarly contaminated sampling sites using PAST V4.17 software. The study area and MP distribution map were prepared using ArcGIS Pro 3.2 software (Esri Inc., USA).

## 3. Results and Discussion

### 3.1. Occurrence and Abundance of MPs

**Figure 2:**
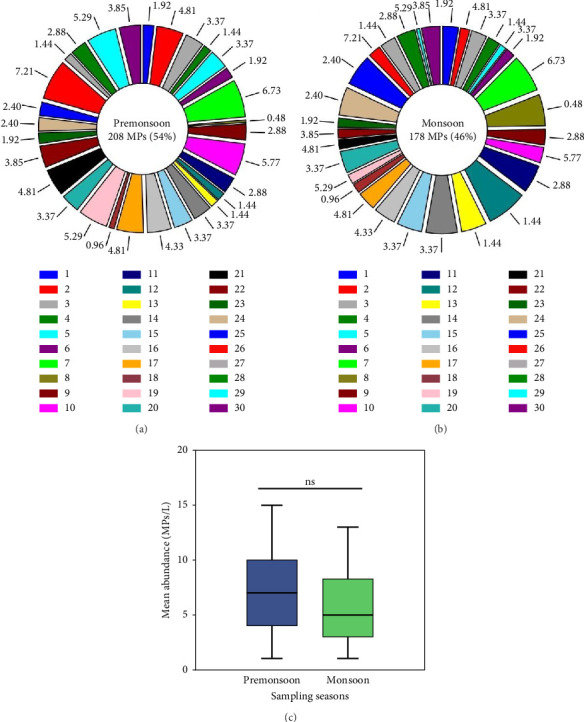
Relative proportion of microplastics during the (a) premonsoon, (b) monsoon season, and (c) seasonal comparison in the surface water of the Jamuna River tested using a Student's *t*-test (*p* > 0.05). Error bars represent ±SD.

MP particles were present in the surface water of all sampling locations. A total of 386 MPs were extracted from the surface water of 30 different stations of the Jamuna River in both seasons. In the premonsoon season, 208 MPs (54%) were observed in the surface water, while 178 MPs (46%) were obtained during the monsoon season (Figures 2(a) and 2(b)). The abundance of MPs in the surface water ranged from 0.01 to 0.15 MPs/L, with a mean of 0.07 ± 0.04 MPs/L in the premonsoon and from 0.01 to 0.13 MPs/L, with a mean of 0.06 ± 0.03 MPs/L in the monsoon season. Accordingly, the student's *t*-test showed that MP abundance had no significant difference between the premonsoon and monsoon seasons (*p* > 0.05; Figure 2(c)).

The mean abundance of MPs in the surface water of the Jamuna River was 0.064 ± 0.03 MPs/L. Results from various coastal places worldwide were compared with the current study's findings (Table 1). It has been observed that the average abundance of MPs in the surface water of the Jamuna River was lower than that of other previously reported surface water concentrations of different rivers across the globe: the Ebro River, Spain [32]; the River Rhine, the Netherlands [33]; the Lambro River, Italy [34]; the River Dalälven, Sweden [35]; the Bay of Brest, France [36]; and the Sarıkum Lagoon, Turkey [37] (Table 1). Conversely, it has been noticed that the mean abundance of MPs in the Jamuna River water was higher than in the Taiwan Strait, Taiwan [38], the Western coast of Australia [39], and offshore, Brazil [40] (Table 1). In addition to comparisons with European rivers, placing our results in the South and Southeast Asian context highlights the regional significance of MP pollution. For instance, Napper et al. [11] reported mean concentrations of 0.02–1.50 MPs/L in the transboundary Ganges River, which are higher than our findings for the Jamuna. Similarly, studies in the Turag and Buriganga rivers in Bangladesh have reported substantial MP loads in surface waters and biota [14, 18]. In India, Ranjani et al. [41] and Velmurugan et al. [42] documented high MP abundance in coastal and riverine systems, while in Southeast Asia, Kieu-Le et al. [43] found significant contamination in the northern branches of the Mekong Delta, Vietnam, and Sulistyowati et al. [44] reported high MP occurrence in the Cisadane River, Indonesia. Chinese rivers such as the Manas River Basin [10] and Wei River [45] also demonstrate concentrations comparable to or higher than our results. These regional studies consistently attribute elevated MP levels to intense urbanization, untreated effluents, mismanaged plastic waste, and textile industry discharges, factors also relevant for the Jamuna River system. By situating our results within this regional framework, our findings gain stronger relevance for South and Southeast Asia, where freshwater systems face similar anthropogenic pressures. The existence of MPs in river water may be caused by a variety of circumstances, such as riverine inputs [4], anthropogenic and tourism-related activities [18], and even the types and chemical makeup of the MPs [46]. Comparing studies from different parts of the world is challenging because there is currently no accepted standard technique. According to Li et al. [47], there is variation in research about the utilization of MP extraction techniques, population density, and anthropogenic pressure in the studied area, and temporal and spatial environmental conditions. It is important to note that cross-study comparisons are limited by methodological differences, including variation in mesh sizes, digestion protocols, and analytical techniques. Therefore, while these comparisons provide context, they should be interpreted with caution.

The nets used for plastic sampling had different mesh sizes. For instance, Simon-Sánchez et al. [32] employed a 5-μm mesh to collect plastics from the Ebro River (Spain), while Campanale et al. [48] used a 330-μm mesh for their survey in the Ofanto River (Southern Italy). The variation in plastic quantities measured can primarily be attributed to the differing mesh sizes, as shown by the negative correlation between the mesh sizes and the relative plastic concentrations recorded in these studies [49]. It is now recommended to use a Manta net with a mesh size of 300–330 μm, in line with the Marine Strategy Framework Directive (2008/56/EC) [49].

The minimum cutoff size for the MP sample and analysis is crucial for such assessments. Furthermore, compared to the monsoon season, the abundance of MP particles in the surface water of the Jamuna River was numerically higher in the premonsoon but not significantly varied (*p* > 0.05). In Bangladesh, there is no or very little rainfall during the premonsoon season, while heavy rain showers are observed during the monsoon season. Therefore, the water volume of the Jamuna River increases by 6 to 7 m on average during the monsoon season. The Jamuna River discharges up to 100,000 m^3^/s, with a bankful discharge rate of about 48,000 m^3^/s, and an average annual water discharge to the Bay of Bengal is about 19,600 m^3^/s [50]. As a result, it causes an increase in the water volume and flow and carries sediments and pollutants like MPs, heavy metals, pesticides, and other contaminants into the Bay of Bengal. Because our sampling used a 63 μm sieve, particles < 100 μm were not captured. Thus, the abundance values reported here should be considered conservative estimates of MP pollution in the Jamuna River.

### 3.2. Spatial Distribution of MPs

**Figure 3:**
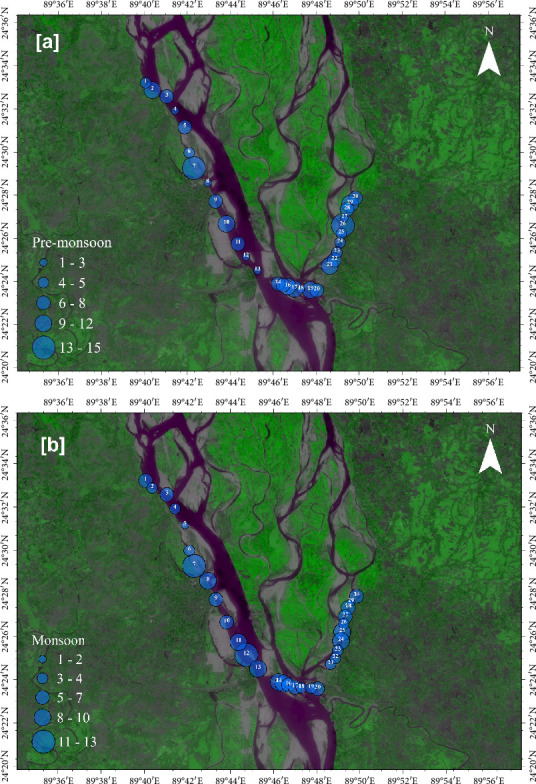
Spatial distribution of MPs in the surface water of the Jamuna River during the (a) premonsoon and (b) monsoon seasons.

In the premonsoon, the highest abundance of MPs was recorded at Station 26 (0.15 MPs/L) and Station 7 (0.14 MPs/L) (Figure 3(a)). The lower abundance of MPs was observed at Stations 8 and 18 accounting for 0.01 and 0.02 MPs/L. On the contrary, the highest MP concentrations were observed at Stations 12 (0.13 MPs/L) and Station 7 (0.12 MPs/L), and lower MP abundance was found at Stations 29 (0.01 MPs/L) and Station 5 (0.02 MPs/L) in the monsoon season (Figure 3(b)).

Higher MP abundance was observed in proximity to hospitals, landfills, textile industries, and ship parking zones. These activities are potential contributors of MPs, as suggested by previous studies [21, 51]. However, our study did not include direct source-tracking analyses, and therefore such linkages remain speculative. Many visitors in the hospital, fishing area, and ship parking zone produce massive amounts of plastic debris daily. Landfill areas are usually the main sources of MPs, and massive amounts of plastic debris with domestic waste are dumped in these areas [51]. Hence, this plastic debris is fragmented and turns into micro- and mesoplastics, contaminating the river water. Textile industries produce a huge amount of plastic fibers, which are transferred to the aquatic environment in different ways [21]. It was noticed that during the monsoon season, river flow is higher than during the premonsoon season due to heavy rainfall in Bangladesh [52, 53] Thus, floating MPs always move from upstream to downstream [54]. Therefore, the exact origin of these MP particles is difficult to predict [9].

**Figure 4:**
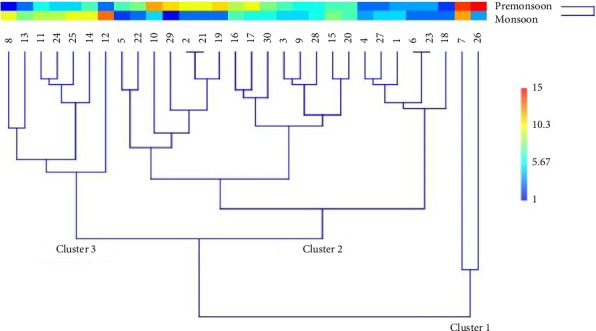
Cluster dendrogram based on similarities of MP distribution across the sampling stations of the Jamuna River.

To further examine the distribution of MP concentrations in the surface water, a hierarchical cluster dendrogram was created using the paired group (UPGMA) Euclidean cluster analysis method (Figure 4). Sampling stations within the same classification were grouped together. The dendrogram revealed three distinct clusters: Cluster 1 includes Stations 7 and 26, and Cluster 2 comprises Stations 1–6, 9–10, 15–23, and 27–30, forming three separate groups with similar pollution distributions under one cluster. The remaining Stations, 8, 11–14, and 24–25, form Cluster 3. Cluster 1 accounts for 7% of the total sampling stations, representing the effluent channel of two textile industries, a general hospital, a ship parking area, and a sand deposit zone. Clusters 2 and 3 represent 70% and 29% of the total sampling stations, respectively.

The spatial distribution of MP particles in the river revealed varying concentrations during the premonsoon and monsoon seasons, with higher and lower levels observed in different areas. It is believed that the presence of several tourist destinations, such as Bangabandhu Jamuna Ecopark, Jamuna Anando Park, and Jamuna Bridge, along with numerous ferry ghats, textile factories, landfills, fishing zones, and agricultural farms on both banks of the Jamuna River, may contribute to the presence of MPs in the surface water of the study area. A previous study found that tourism activities in the remote areas around Mahodand Lake in Pakistan led to higher MP concentrations [55]. Similarly, the significant number of MPs (5000–757,500 MPs/km^2^) found in China's Qinghai Lake highlights how MP pollution is particularly concentrated near tourist attractions [56].

### 3.3. Shape Distribution of MPs

**Figure 5:**
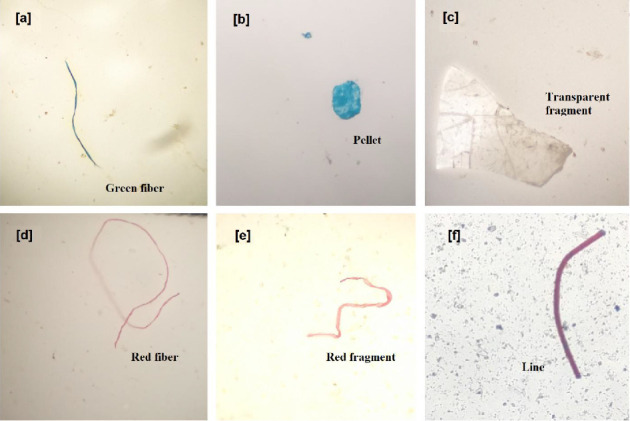
Photographs of different microplastic shapes observed under × 4 and × 10 magnifications. (a) green fiber, (b) pellet, (c) transparent fragment, (d) red fiber, (e) red fragment, and (f) line-shaped microplastic particles.

MPs found from all the analyzed surface water samples were found to be irregularly shaped and sorted into fragments, foam, fiber, lines, and pellets (Figure 5). Fibers and fragments were the dominant shapes of MPs found in the surface water of the Jamuna River in both seasons, constituting about 35.58% and 29.33% in the premonsoon and 60.11% and 17.42% of the total forms of MPs in the monsoon season. Findings of this study are consistent with the worldwide reported data [57]. It is important to remember that compared to other forms of MPs, fragments and fibers are typically more harmful [58]. Thus, it has been hypothesized that the higher frequency of fibers and fragments and the corresponding decrease in diversity could raise the danger of MPs. Additionally, it has been proposed that some of the fibers found in the samples may be able to go farther due to air fallout. On the other hand, fibers that usually come loose from synthetic textiles originate from metropolitan regions [59].

**Figure 6:**
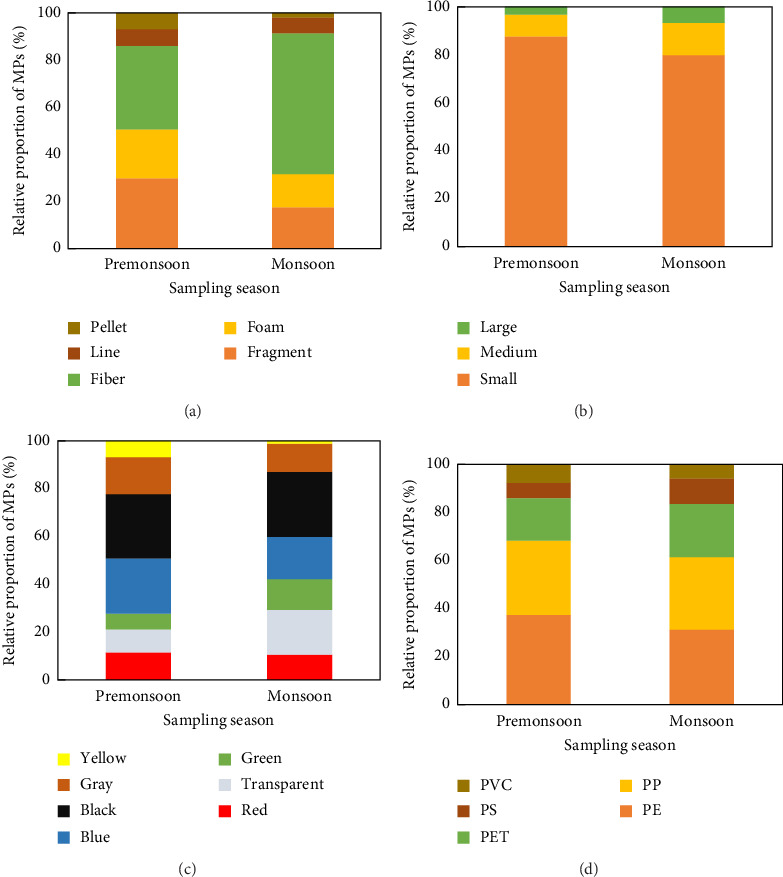
(a) Proportion of different MP shapes (pellets, lines, fibers, foam, and fragments) in surface water during premonsoon and monsoon. (b) Seasonal variation in MP colors, showing dominance of black and blue in premonsoon and black and transparent in monsoon. (c) Size class distribution of MPs (100–1500 μm, 1500–3000 μm, 3000–5000 μm) during both seasons. (d) Polymer composition of MPs (PE, PP, PET, PS, and PVC) in surface water during premonsoon and monsoon.

The other three shapes of MPs, such as line, indicated a similar proportion (7.21% and 6.74%), foam (20.67% and 13.48%), and pellet (7.21% and 2.25%) in the premonsoon and the monsoon, respectively (Figure 6(a)). The result mentioned above indicated that the shape of the MPs was principally fiber-based in the surface water of the Jamuna River. The proportion of fibers was higher in the monsoon than in the premonsoon season (*p* < 0.05). The greater concentrations of fibers may have come from a wide range of sources, including airborne MPs, laundry, domestic wastewater, fishing gear, and industrial fisheries, among other things [60]. In contrast, Wang et al. [10] found that the dominant shape of MP was fiber in April (78.76%) and July (86.87%). The irregularly shaped fragment was the second largest type in the observed water samples in both seasons, resulting from the breakup of thicker plastic products such as bottles, plastic strips, bags, and other items.

### 3.4. Color Distribution of MPs

In addition to possibly containing hazardous colorants that endanger aquatic life, MP's coloration frequently reveals its origins. The MPs identified in the water samples had a range of colors, and the identified colors were red, transparent, green, blue, black, yellow, and grey. The literature has reported that black, blue, translucent, and white MPs made up 75.3% of the overall color diversity, with an average of 0.69 [57], which was like our findings. This study revealed that black and blue (27.40% and 22.60%) colored particles were the dominant colors in the sampled water during the premonsoon and black and transparent (27.53% and 18.54%) in the monsoon season, respectively (Figure 6(b)).

The presence of these MPs may be attributed to the widespread use of plastic film in agriculture or could stem from tire wear and discharges from wastewater treatment plants, which are significant sources of MPs [57, 61]. Plastic remnants were found in both soil and surface water, partly due to the black color of the mulch commonly used [62]. The variety of MP particle colors observed in the surface water of the Jamuna River can be linked to textile effluents mixing with the water. Effluent from the textile and cotton industries contains large amounts of MP particles [21]. Additionally, the aging of these materials in aquatic environments may explain the significant increase in brown and yellow MPs. For example, Hu et al. [63] found that plastics exposed to UV light aged and turned yellow, while Zhang et al. [64] observed visible browning in MPs from fertilizer samples.

However, other colored plastics were found in relatively low but varying proportions in each season. The findings above showed that the color of MPs is similar between the two seasons, with different proportions. However, this did not indicate that the source of pollution was the same in all cases. Notably, it was related to the shape of MPs [10]. Additionally, environmental conditions during transmission cause the color of the MPs to fade, changing the corresponding polymer's color, which may also result in a high proportion of MPs with the same color between the two seasons [65]. Therefore, color can be examined to measure the origin of MPs. Black and blue MPs constitute a significant percentage of the studied water, originating from background contamination such as household waste and paint flakes. Red, green, and yellow were also identified, and colored plastic goods like clothing and bags may be the primary source of multicolored MPs.

### 3.5. Size Distribution of MPs

In this study, MPs were categorized into three size groups: (i) small MPs (100–1500 μm); (ii) medium MPs (1500–3000 μm); and (iii) large MPs (3000–5000 μm). The results showed that small particles were the most prevalent in the surface water of the Jamuna River, comprising 87.98% during the premonsoon and 79.78% during the monsoon seasons (Figure 6(c)). Particle size is closely related to the potential hazards posed by MPs, with smaller particles generally presenting a greater risk [66]. As such, particle size distribution was a key focus of this study. A global meta-analysis of freshwater ecosystems across 36 countries by Chang et al. [57] found that small MPs made up an average of 69% of all freshwater MPs. Moreover, there is a strong correlation between MP abundance and size, with smaller MPs typically being more abundant [67].

However, the other two classes, that is, the 1500–3000 μm and the 3000–5000 μm classes, accounted for a smaller percentage, only 8.65% and 3.37% of the observed MPs in premonsoon and 13.48% and 6.74% in the monsoon (Figure 6(c)). In the present study, small MPs (100–1500 μm) were most abundant, indicating that large plastics fragment into smaller MPs due to environmental and human factors, and as their sizes decrease, the number of small MPs will rise significantly over time [68]. Also, the size of MP particles observed in water samples did not vary significantly between the premonsoon and the monsoon seasons, which indicates that tiny MPs persist in the water for a long time and constantly affect the ecological environment [66]. Previous investigations have shown that smaller MPs typically have a higher bioavailability and can build up in an organism's cells more easily [69].

### 3.6. Identification of Polymer Types

**Figure 7:**
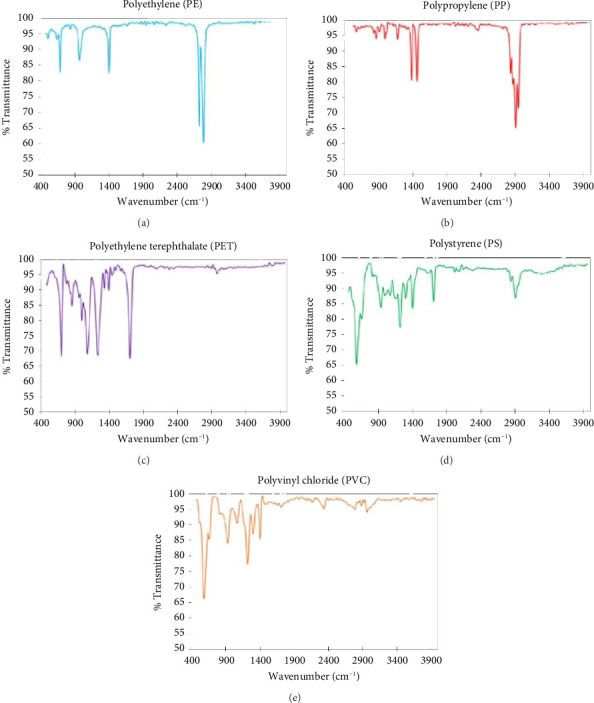
FTIR spectroscopy peaks in polymers (a) polyethylene (PE), (b) polypropylene (PP), (c) polyethylene terephthalate (PET), (d) polystyrene (PS), and (e) polyvinyl chloride (PVC) present in surface water samples in the Jamuna river, Bangladesh.

The MP samples collected on filter membranes were analyzed using FTIR spectroscopy to identify various polymer types in surface water samples. Five polymers were identified: PE, PP, PET, PS, and PVC (Figure 7). Each polymer displayed characteristic FTIR peaks: PE showed stretching and bending vibrations at 2916 cm^−1^ and 1465 cm^−1^; PP had distinct C-H stretching and CH_2_/CH_3_ bending vibrations around 2950 cm^−1^ and 1460 cm^−1^; PET exhibited C=O stretching around 1720 cm^−1^ and aromatic ring vibrations at 1500 cm^−1^; PS revealed aromatic C-H and C=C stretching vibrations around 3026 cm^−1^ and 1601 cm^−1^; PVC displayed C-H and CH_2_ bending vibrations at 2960 cm^−1^ and 1426 cm^−1^ [70–73]. These FTIR spectra allowed for the identification and characterization of these common MP polymers.

### 3.7. Abundance and Sources of Polymers in the Study Area

This study revealed that PE and PP were the most prevalent polymers in the sample studied. The mean abundance of PP, PE, PS, PET, and PVC was 30.34%, 37.24%, 6.71%, 18.43%, and 7.28%, respectively, during the premonsoon season and 28.72%, 31.60%, 10.81%, 23.23%, and 5.64%, respectively, during the monsoon season (Figure 6(d)). The findings of this study were consistent with the world's plastic statistics. As per Plastics Europe [74], the PP, PE, and PET polymers in the overall plastic demand were 19.40%, 29.80%, and 7.90%, respectively. According to earlier research, the most common polymer kinds of MPs in the aquatic environment were PP, PE, and PET [4].

These plastics are usually used in the production of packaging materials, clothing, and products for commercial composting, all of which have short lifespans and low recycling rates, making them major contributors to household waste [1]. However, the primary sources of various polymer types of MPs in aquatic environments have been explored in existing literature [1, 18, 75–79]. For example, Yan et al. [79] found that PE is widely used in fishing lines, nets, and ropes due to its strength, durability, flexibility, water resistance, and buoyancy [1, 18]. Additionally, PE's widespread use in the fishing industry and in various industrial applications likely contributes to its high prevalence in the surface water of the study area, with an average of 34.42% of MPs in the Jamuna River.

PP was the second most common polymer found in the MPs in the Jamuna River, with an average of 29.53%. The lightweight nature and high tensile strength of PP make it ideal for fishing nets, ropes, and twines, which are easy to handle and durable in harsh aquatic environments [79]. Given that around 6445 metric tons of fish are caught annually in the Jamuna River [23], the extensive use of fishing lines, nets, and ropes contributes significantly to the presence of PP and PE in the river. PET is commonly used for beverage bottles due to its strength, lightness, and clarity [75]. While the exact number of plastic bottles used in Bangladesh is unknown, it is estimated that about 500 billion plastic bottles are used each year, with many PET bottles ending up in the environment. The Jamuna River, flowing through multiple cities and industrial areas, receives a significant influx of PET bottles, contributing to MP pollution (PET: average 20.83%) as they degrade or break down through improper disposal, runoff, and industrial discharges [4]. PET's use in packaging, clothing, and composting further exacerbates the issue, as these products have short life cycles and low recycling rates [77].

Surface water pollution by MPs is worsened by the widespread use of PS. Found in items like disposable food containers, packaging materials, and insulation, PS is prone to fragmentation due to its brittle nature and low resistance to environmental stressors [75]. These fragments often enter surface waters through littering, improper waste management, and runoff [79]. The Jamuna River in Bangladesh, popular for its scenic beauty and cultural experiences, attracts many visitors each year. Tourists engage in boating, picnicking, and sightseeing, while local festivals and events increase the flow of disposable containers, packaging materials, household items, and PS fragments into the river. Likewise, PVC, used in pipes, drainage systems, flooring, window frames, medical devices, and fishing equipment, also contributes to MP pollution when it degrades or breaks down [75, 79]. PVC's leaching additives further exacerbate environmental hazards, making it a significant contributor to MP contamination in the Jamuna River.

### 3.8. Risk Assessment of MPs

#### 3.8.1. PHI

The PHI, calculated based on five identified polymers, indicated a hazard Category IV (“Danger” risk level) during both the premonsoon (PHI_ZONE_ = 305.65) and monsoon seasons (PHI_ZONE_ = 208.0) (Table 2). These PHI values are concerning, highlighting the potential risks posed by MPs in aquatic ecosystems. MPs are known to negatively impact both human health and the environment [24]. Notably, PVC and PS exhibited particularly high hazard scores, suggesting that their presence in aquatic environments could have harmful effects on aquatic life.

MPs have the ability to absorb, carry, and leach chemicals such as bisphenol A and POPs [80]. The adsorption capacity of MPs in aquatic environments was clarified by several studies [81]. The health of aquatic animals may be seriously threatened by MP-contaminated fish feed. When evaluating the danger of MPs, PHI is a crucial factor. The high hazard scores of PS (hazard score = 30) and PVC (hazard score = 10001) in the current investigation resulted in a significantly elevated PHI value [28]. Fish treated with MP-contaminated feed experienced higher cell death and DNA damage in their liver tissues after prolonged exposure to propylene [82].

#### 3.8.2. PLI

**Figure 8:**
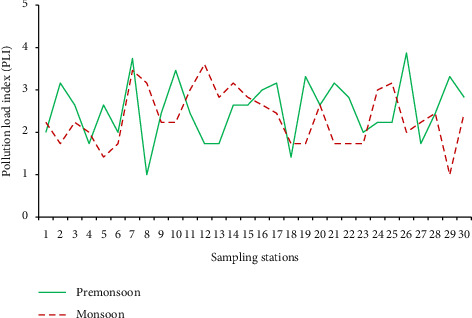
Estimated pollution load index (PLI) of all sampling stations for premonsoon and monsoon seasons.

The PLI value ranged between 1.00 and 3.87 during the premonsoon and 1.00 and 3.61 during the monsoon season (Figure 8). The level of MP pollution in a specific location is measured using PLI. The PLI can be divided into four groups, which correspond to risk categories I, II, III, and IV: PLI < 10, PLI 10–20, PLI 20–30, and PLI > 30 [78]. In the present study, all the surface water samples have a PLI value of less than 10 for the premonsoon and monsoon seasons, indicating risk Category I. According to Ranjani et al. [41], the PLI values from Maharashtra, Karnataka, and Lake Vembanad were 15.5, 11.4, and 10.45, respectively. These values are significantly higher than our current study and are considered hazard level II on Indian coasts. Changjiang Bay had a higher average PLI value than the Indian coast and our current study, at 18.4 [78].

#### 3.8.3. PERI

The PERI values of the Jamuna River surface water show high ecological risk. The PERI value was estimated at 2454.43 during the premonsoon and 1911.29 during the monsoon seasons from combined MP polymers in the surface water of the Jamuna River. The present study revealed that the surface water of the Jamuna River is under the “extreme danger” risk category (PERI > 1200). Fardullah et al. [83] reported a PERI value of 14722 for the surface water of Hatiya Island, Bay of Bengal, much higher than our present study. The hazard score and MP polymer abundance are used to calculate PERI. For instance, high PERI values were found in the Jamuna River due to the significant concentration of PVC, PE, and PS. The initial ecological risk assessment resulting from MP pollution in the surface water of the Jamuna River in Bangladesh was presented in this study by the combined use of PHI, PLI, and PERI values. It is important to note that the present study is based on two seasonal snapshots, which may not fully capture interannual or temporal variability in MP pollution. Future studies incorporating multi-year sampling and greater replication are required to confirm the trends reported here and to provide a more comprehensive risk assessment.

The discrepancy between PLI and PERI reflects fundamental differences in these indices. While PLI is based solely on relative abundance, PERI integrates both abundance and toxicity coefficients of specific polymers. In the Jamuna River, MP abundance was relatively low, explaining the minor pollution classification (PLI). However, the dominance of high-hazard polymers such as PVC and PS elevated the PERI to the “extreme danger” category. Thus, PERI provides a more ecologically relevant assessment in this context, as it accounts for polymer toxicity in addition to abundance. Despite these limitations, the study provides important baseline data on MP pollution in the Jamuna River and highlights priority areas for future research, including high-resolution year-round monitoring, advanced source tracking, and improved detection of smaller particle sizes.

## 4. Conclusion

The study revealed the widespread MP pollution in the Jamuna River, highlighting the significant accumulation from industrial, hospital, and urban waste sources. Findings indicate that MPs are prevalent and consist mainly of harmful plastic types such as PE, PS, and PVC, which are known to pose ecological risks. These small particles and chemical properties enable them to persist in aquatic environments, potentially bioaccumulating in the food web. This study revealed that all the surface water samples have a PLI value of less than 10 for premonsoon and monsoon seasons, indicating risk Category I. Subsequently, the PHI value highly increased due to the high hazard score of PS (hazard score = 30) and PVC (hazard score = 10001). The study also suggested that the surface water of the Jamuna River is under the “extreme danger” risk category (PERI > 1200), considering the hazard score and abundance of MP polymers. This research not only provides the first systematic risk assessment of MP contamination using PHI/PLI/PERI in the Jamuna River but also demonstrates how its hydrological dynamics and polymer-specific risk profiles differ from other Bangladeshi river systems. A key limitation of this study was the absence of year-round sampling, which may have hindered a full understanding of seasonal variations and long-term trends in MP pollution. Therefore, our ecological risk assessment should be regarded as preliminary. Long-term, high-resolution monitoring with greater replication will be essential to validate and expand upon these findings. To address this, future research should incorporate more comprehensive, year-round sampling to capture temporal fluctuations and better assess the cumulative risks posed by MPs in freshwater ecosystems. Although this study advances understanding of MP distribution in the Jamuna River, source attribution remains speculative without direct data or modeling, and future tracer or modeling approaches are needed to confirm specific anthropogenic sources. Additionally, the exclusion of particles smaller than 100 μm and the use of FTIR confirmation on subsets may have underestimated total abundances and diversity of polymer types. Nonetheless, this research provides valuable baseline data that can inform mitigation strategies to manage MP contamination in the region [84, 85].

## Figures and Tables

**Table 1:** Concentrations of microplastics (items/m^3^) were measured in some European watercourses.

Watercourse	Country	Items (m^3^)	Filtration mesh (μm)	Polymer detection method	References
Western Coast of Australia	Australia	0.02	333	Microscope	Reisser et al. [39]
Northeast coast	Brazil	0.01	300	Microscope	Ivar do Sul and Costa [40]
River Dalälven	Sweden	4.5	330	NIR	Van der Wal et al. [35]
Lambro River	Italy	0.4 ± 0.2–14.3 ± 11.0	300	ATR–FTIR	Magni et al. [34]
Danube River	Romania	10.6	330	ATR–FTIR	Van der Wal et al. [35]
River Rhine	The Netherlands	1.85–4.92	330	NIR	Van der Wal et al. [35]
River Rhine	The Netherlands	1.85–4.92	330	ATR–FTIR	Van der Wal et al. [35]
Sarıkum Lagoon	Turkey	2.67 ± 2.33	300	FTIR	Öztekin and Bat [37]
Bay of Brest	France	0.24 ± 0.35	335	FTIR	Frère et al. [36]
Sarıkum Lagoon	Turkey	2.67 ± 2.33	300	ATR–FTIR	Oztekin and Bat [37]
Taiwan Strait	Taiwan	0.026	330	μATR–FTIR	Wu et al. [38]
Ofanto River	Italy	0.9 ± 0.4–13 ± 5	333	Py–GC/MS	Campanale et al. [48]
Lambro River	Italy	0.4 ± 0.2–14.3 ± 11.0	300	μATR–FTIR	Magni et al. [34]
Manas River Basin	China	21 ± 3–49 ± 3	153	μ-FTIR	Wang et al. [86]
Buriganga River	Bangladesh	4.33 ± 0.58–43.67 ± 0.58	300	ATR–FTIR	Islam et al. [22]
Cisadane River	Indonesia	13.33–113.33	200	FTIR	Sulistyowati et al. [44]
Meghna Estuary	Bangladesh	33.33–316.67	300	FTIR	Hossain et al. [87]
Wei River	China	3.67–10.7	75	Microscope MV5000 (R/TR)	Ding et al. [45]
Karnaphuli River	Bangladesh	14.24–26.68	330	FTIR	Jui et al. [88]
Mekong River Delta	Vietnam	6.0 ± 2.0	250	FTIR–ATR	Kieu-Le et al. [43]
Karnafully River	Bangladesh	0.57 ± 0.07 to 6.63 ± 0.52	20	μ-FTIR	Hossain et al. [21]
East Coast, from Ennore to Kovalam	India	6–30	300	AIR	Velmurugan et al. [42]
Surma River	Bangladesh	5–20	20	FTIR	Rahman et al. [89]
Urban surface waters of Wuhan	China	1660.0 ± 639.1 to 8925 ± 1591	50	Microscope	Wang et al. [90]
Lahore Canal & Drains	Pakistan	92.4 ± 0.3 to 29.9 ± 0.1	Mist net (8 m long × 60 cm high)	FTIR	Mehboob et al. [91]
Guangdong-Hong Kong-Macao Greater Bay Area	Hong Kong	25.5 ± 3.5	333	μATR–FTIR	Wu et al. [92]
Meishe River	China	3–10	300	μ-FTIR	Wen et al. [93]
Jamuna River	Bangladesh	0.01 ± 0.07 to 0.15 ± 0.04	300	FTIR	Present study

*Note:* The water sampled, a mesh of the nets, and the equipment for plastic identification are also shown. μATR–FTIR = FTIR attenuated total reflection–micro-spectroscopy; ATR–FTIR = FTIR attenuated total reflection spectroscopy.

Abbreviations: FTIR, Fourier-transform infrared spectroscopy; NIR, near-infrared spectroscopy; Py–GC/MS, pyrolysis–gas chromatography/mass spectrometry.

**Table 2:** Polymers identified in the present study are classified based on hazard level and polymer hazard index (PHI) and potential ecological risk index (PERI).

Polymer type	Monomer	Hazard	Hazard score	PHI			PERI		
				Premonsoon	Monsoon		Premonsoon	Monsoon	
Polyethylene (PE)	Ethylene	Extremely flammable gas may cause drowsiness/dizziness, respiratory irritation	11	2.31	2.86	Hazard Category: IV (100–1000) Risk category: danger	13.65	11.59	Hazard Category: V (> 1200) Risk category: extreme danger
Polypropylene (PP)	Propylene	Extremely flammable gas, harmful when inhaled	1	0.43	0.38		1.01	0.96	
Polyvinyl chloride (PVC)	Vinyl chloride	May cause cancer	10001	300.03	200.02		2426.91	1880.19	
Polyethylene terephthalate (PET)	Ethylene terephthalate	Harmful if swallowed	4	1.08	0.84		6.14	7.74	
Polystyrene (PS)	Styrene	Acute toxic	30	1.8	3.9		6.71	10.81	
				PHI_ZONE_ = 305.65	PHI_ZONE_ = 208.0		PERI_ZONE_ = 2454.43	PERI_ZONE_ = 1911.29	

## Data Availability

The data that support the findings of this study are available on request from the corresponding author. The data are not publicly available due to privacy or ethical restrictions.

## Supporting Information

Table S1: Geographical coordinates and mean abundance (±standard deviation) of the analyzed surface water sample in the Jamuna River, Bangladesh.

Table S2: Indices are used to describe the hazard level of microplastic pollution.
