# Supplementary material for: Potential Ecological Risk and Characterization of Floating Microplastics in the Surface Water of a Highly Urbanized Large River in Southeast Asia
Source: Scientifica (Cairo). 2025 Oct 8;2025:3043345. doi: 10.1155/sci5/3043345 (PMC12527606; doi:10.1155/sci5/3043345)
Supplement: Supporting Information — Additional supporting information can be found online in the Supporting Information section. [file 3043345.f1.docx]

**Supplementary Information**

**Table S1.** Geographical coordinates and mean abundance (± Standard deviation) of the analyzed surface water sample in the Jamuna River, Bangladesh

| **Sample**  **site** | **GPS reading** | | **Surrounding information at each**  **sampling points in the Jamuna River** | **Abundance (MPs/L)** | |
| --- | --- | --- | --- | --- | --- |
|  | **Latitude** | **Longitude** |  | **Pre-monsoon** | **Monsoon** |
| 1 | 24.392360 | 89.770120 | Cotton factories and farmland | 0.04 ± 0.01 | 0.05±0.02 |
| 2 | 24.399891 | 89.770111 | Landfill | 0.10 ± 0.03 | 0.03±0.01 |
| 3 | 24.465527 | 89.717892 | Rural children park | 0.07 ± 0.02 | 0.05±0.02 |
| 4 | 24.452120 | 89.725610 | Gas station and farmland | 0.03 ± 0.01 | 0.04±0.01 |
| 5 | 24.422170 | 89.737810 | Small industries and farmland | 0.07 ± 0.01 | 0.02±0.01 |
| 6 | 24.422360 | 89.738030 | Cotton factories | 0.04±0.01 | 0.03±0.01 |
| 7 | 24.422120 | 89.738120 | Sirajganj General Hospital | 0.14±0.04 | 0.12±0.03 |
| 8 | 24.421000 | 89.739290 | Cargo and ship parking zone | 0.01±0.01 | 0.10±0.03 |
| 9 | 24.420120 | 89.739120 | Chaina Badh Tourist spot | 0.06±0.02 | 0.05±0.01 |
| 10 | 24.407468 | 89.751007 | Sirajganj Economic Zone | 0.12±0.03 | 0.05±0.02 |
| 11 | 24.407468 | 89.751007 | Bangabandhu Jamuna Eco Park | 0.06±0.01 | 0.09±0.02 |
| 12 | 24.397580 | 89.765720 | Fishing activities and landfill | 0.03±0.01 | 0.13±0.04 |
| 13 | 24.401920 | 89.768570 | Agricultural activities | 0.03±0.01 | 0.08±0.02 |
| 14 | 24.398405 | 89.769524 | Power grid company | 0.07±0.02 | 0.10±0.01 |
| 15 | 24.396590 | 89.768930 | Fishing activities | 0.07±0.01 | 0.08±0.02 |
| 16 | 24.395120 | 89.773120 | Fishing hotspot area | 0.09±0.02 | 0.07±0.01 |
| 17 | 24.393000 | 89.776120 | Fishing and agricultural activities | 0.10±0.03 | 0.06±0.02 |
| 18 | 24.396000 | 89.802600 | Construction and ship parking zone | 0.02±0.01 | 0.03±0.01 |
| 19 | 24.401000 | 89.802120 | Jamuna Agro Farm | 0.11±0.04 | 0.03±0.01 |
| 20 | 24.391200 | 89.792120 | Jamuna Resort landfill | 0.07±0.01 | 0.07±0.02 |
| 21 | 24.390710 | 89.770131 | Cantonment landfill | 0.10±0.02 | 0.03±0.01 |
| 22 | 24.494440 | 89.713800 | Domestic effluent and dumping area | 0.08±0.01 | 0.03±0.01 |
| 23 | 24.495570 | 89.713450 | Fishing and agricultural activities | 0.04±0.01 | 0.03±0.01 |
| 24 | 24.496330 | 89.713570 | Construction farm and military camp | 0.05±0.02 | 0.09±0.02 |
| 25 | 24.497140 | 89.713480 | Gas station and farmland | 0.05±0.01 | 0.10±0.03 |
| 26 | 24.406244 | 89.798244 | Sand deposit and ship parking zone | 0.15±0.04 | 0.04±0.01 |
| 27 | 24.408482 | 89.806446 | Nikrail Union landfill site | 0.03±0.01 | 0.05±0.01 |
| 28 | 24.399530 | 89.776853 | Blacksmith village | 0.06±0.01 | 0.06±0.02 |
| 29 | 24.399530 | 89.776854 | Cotton factories and a small market | 0.11±0.03 | 0.01±0.01 |
| 30 | 24.398530 | 89.775828 | Fertilizers factories | 0.08±0.02 | 0.06±0.01 |

**Table S2**.  Indices are used to describe the hazard level of microplastic pollution.

| **PHI** | **Hazard category** | **PLI** | **Hazard category** | **PERI** | **Risk category** |
| --- | --- | --- | --- | --- | --- |
| 0–1 | I | < 10 | I | <150 | Minor |
| 1–10 | II | – | – | 150–300 | Moderate |
| 10–100 | III | 10–20 | II | 300–600 | High |
| 100–1000 | IV | 20–30 | III | 600–1200 | Danger |
| >1000 | V | > 30 | IV | >1200 | Extreme danger |
